# The prevalence, characteristics, and psychological wellbeing of unpaid carers in the United Kingdom

**DOI:** 10.1007/s00127-024-02745-8

**Published:** 2024-08-10

**Authors:** Enya Redican, Richard Meade, Craig Harrison, Orla McBride, Sarah Butter, Jamie Murphy, Mark Shevlin

**Affiliations:** 1 School of Psychology, Ulster University, Cromore Road, Coleraine, NI BT52 1SA UK; 2 Carers NI, Belfast, UK; 3 Carers Scotland, Glasgow, UK

**Keywords:** Informal carers, Anxiety, Depression, Help-seeking, Prevalence

## Abstract

**Background:**

This study sought to describe the characteristics of unpaid carers in the UK and assess levels of depression, anxiety, and mental health treatment seeking behaviours in this population.

**Methods:**

Data was derived from Wave 9 (n = 2790) of the COVID-19 Psychological Research Consortium (C19PRC) study, a longitudinal survey of adults in the UK. Logistic regression analyses were conducted to examine the characteristics of unpaid carers, association between caregiver status and psychological wellbeing, and caregiver-specific factors associated with risk of poor psychological wellbeing.

**Results:**

Approximately 15% (n = 417) of the sample reported providing unpaid care. Younger age, having three or more children in the household, and lower income were identified as significant correlates of caregiver status. Unpaid caregivers were at increased risk of depression or anxiety and mental health help-seeking. Unpaid caregivers who were younger, lived in households with one or two children, and had a lower income were at greater risk of depression or anxiety and engaging in mental health help-seeking. Caring for an individual with a terminal illness, long-term illness, learning disability or difficulty, mental health problems, physical disability, and other were linked to increased risk of depression or anxiety, while caring for someone with a learning disability increased risk of mental health help-seeking.

**Conclusions:**

This study indicates that at least one in eight people in the UK provide unpaid care, and that those who provide unpaid care have a far higher risk of experiencing depression or anxiety and seeking mental health treatment. The identification of risk factors associated with these mental health outcomes will facilitate the identification of those in most need of support.

**Supplementary Information:**

The online version contains supplementary material available at 10.1007/s00127-024-02745-8.

## Introduction

The world’s population is ageing rapidly. By 2050, one in six people are projected to be 65 years or older, up 8% from 2019, and the proportion of people who are 80 years or older is anticipated to triple [1]. The ageing population will have a major impact on many facets of society, including increased demands for health and social care [2]. As a result, the need for unpaid care—care provided by family, friends, or neighbours—will rise dramatically. Smaller families, more women in the workforce, and later retirement ages will all add to this growing demand [3, 4]. Currently, unpaid caregiving is highly prevalent in the population. In the United Kingdom (UK), it is estimated that over 5.7 million people (or 9.3% of the UK population) are providing unpaid care for family members or friends with long-term physical or mental health conditions, or issues related to old age [5, 6]. This figure will only continue to increase in the upcoming years.

Although unpaid caring has many benefits, such as increasing one’s self-esteem, promoting personal development, and enhancing relationships with care recipients [7, 8], it has mostly been associated with negative effects on physical and mental health [9–11]. Most significant adverse health consequences linked to unpaid care are confined to mental health issues [12]. Two of the most common mental health issues experienced by unpaid carers are depression and anxiety [12–14]. However, not all carers experience these negative impacts on their mental health; this is likely due to a combination of demographic and caring-specific factors. Research has shown how females, married carers, and those in full-time employment are more likely to experience poor mental health [9, 15]. Research has also shown how the illness of the care recipient plays a role in determining mental and physical health [16, 17]. Furthermore, those who provide a higher intensity of caring experience worse health outcomes [9, 15] as well as those caring for a spouse or child as compared to parents or other [18]. Overall, the extant evidence base suggests that there are subgroups of carers who experience worse mental health than others. Currently, there are interventions that carers can use to address mental health challenges such as respite care. However, the identification of those subgroups of carers who experience such maladaptive outcomes is essential for the development of targeted and effective interventions [9].

Consequently, the main objectives of the current study were to describe the unpaid caring population and to identify factors associated with an increased likelihood of depression, anxiety, and mental health help-seeking in a large, nationally representative UK sample. Based on existing research, it was anticipated that significant correlates of caring status would include female gender, being a middle-aged adult, and being unemployed [19]. Moreover, based on the extant evidence base, it was anticipated that significant correlates of depression or anxiety and mental health help-seeking would include both demographic (i.e., female gender, being married, being in employment) and caregiving-specific factors (i.e., providing a higher intensity of caregiving, providing care for a spouse or child, providing care for those with disorders such as dementia or cognitive impairment or mental illness).

## Methods

### Participants

Data for the current study was derived from Wave 9 of the COVID-19 Psychological Research Consortium (C19PRC) Study which was established in March 2020 to assess the long-term psychological, social, and economic impact of the COVID-19 pandemic on the UK general population [20]. The C19PRC Study was launched in March 2020, with over 2,025 adults completing Wave 1 of the survey. The survey company *Qualtrics* was used to recruit participants for Wave 9. The Wave 9 study comprised of recontacts (from earlier waves), in addition to Phase 1 and Phase 2 top-ups. Data collection for Wave 9 of the C19PRC was conducted between 24th March and 14th August 2023. The final sample comprised of 2790 participants. Just over half of participants were female (50.4%; n = 1401), and more than two-thirds were employed (62.0%; n = 1729). Most of the sample identified as white British or Irish (87.7%; n = 2405), and the gender distribution was relatively consistent across the different age categories. Further detailed sample characteristics are included in Supplementary Table 1.

### Measures

#### Depression or anxiety

Depression was measured using the *Patient Health Questionnaire-9* [PHQ-9; 21] and anxiety was measured using the *Generalized Anxiety Disorder 7-item Scale* [GAD-7; 22]. Both scales require participants to indicate over the last 2 weeks, how often they have been bothered by any of the relevant problems using a four-point scale ranging from ‘Not at all’ (0) to ‘Nearly every day’ (3). Possible scores on the PHQ-9 range from 0 to 27, and on the GAD-7 from 0 to 21, with higher scores indicating higher levels of depression and anxiety, respectively. Scale scores of 10 or greater are typically used to indicate clinical caseness on each of these measures [21, 22]. In the current study, internal reliability of the PHQ-9 (α = 0.94) and the GAD-7 (α = 0.96) were high. For the purposes of the current study, participants were categorised as having depression or anxiety if they had probable diagnostic status for depression and/or anxiety.

#### Mental health help-seeking

Participants were provided with the following information: *“Mental health difficulties are very common*. *It will help us understand our survey results if you would tell us whether you currently or have in the past received treatment (medication or talking therapies) for these kinds of difficulties*.*”* Options were provided, of which the participants were required to tick which statement applies: “(1) I have never received treatment for mental health problems, (2) I have received treatment for mental health problems in the past, (3) I am currently receiving treatment for mental health problems, (4) I am currently on a waiting list to receive treatment for a mental health problem, and (5) Prefer not to answer”. A binary variable was created to represent current mental health help seeking with option 3 being considered as current mental health help seeking and all other options being considered as not currently engaging in mental health help seeking.

#### Demographic variables

Participants were asked to report their gender (male, female, transgender, prefer not to say, other) and age (in years). For the purposes of the survey, gender was recoded into a binary variable (0 = male, 1 = female), with the other gender responses recoded and treated as missing data. Age was recoded into a multi-category variable (0 = 18–24 years, 1 = 25–34 years, 2 = 35–64 years, 3 = 65 years and over). Participants were asked to indicate their legal marital or same-sex civil-status using 11 categories (1 = single-never married, 2 = Married, 3 = Cohabiting, 4 = Separated, still legally married, 5 = Divorced, 6 = Widowed, 7 = In a registered same-sex civil partnership, 8 = Separated but still legally in a same-sex civil partnership, 9 = Formally in a same-sex civil partnership which is now legally dissolved, 10 = Surviving partner from a same-sex civil partnership, and 11 = In a relationship but not living together). For the current study, a binary variable was created representing relationship status by collapsing options 2, 3, 7, and 11 (Yes = 1; No = 0). Participants were asked to “*Please choose from the following options to indicate your approximate gross (before tax is taken away) household income in 2022*. *Include income from partners and other family members living with you and all kinds of earnings including salaries and benefits”*. Options included one of five categories: “£0–£300 per week (equals about £0–£1290 per month or £0–15,490 per year)”, “£301–£490 per week (equals about £1,291–£2,110 per month or £15,491–£25,340 per year)”, “£491–£740 per week (equals about £2,111–£3,230 per month or £25,341–£38,740 per year)”, “£741–£1,111 per week (equals about £3,231–£4,830 per month or £38,741–£57,930 per year)”, and “£1,112 or more per week (equals about £4,831 or more per month or £57,931 or more per year)”. Employment status was measured using nine categories (1 = employed full-time, 2 = employed part-time, 3 = self-employed full-time, 4 = self-employed part-time, 5 = unemployed, looking for work, 6 = unemployed, family or home, 7 = unemployed, sick or disability, 8 = retired, 9 = full-time student). Employment status was measured a binary variable (0 = unemployed (options 5,6,7,8,9), 1 = employed (options 1,2,3,4)).

#### Household-related factors

Participants were asked “How many adults (18 years or above) live in your household (including yourself)?”. A multi-category variable was created (1 = one adult, 2 = two adults, 3 = three adults, 4 = four or more adults). Similarly, participants were asked “How many children (below the age of 18) live in your household?”. A multi-category variable was also created (0 = no children, 1 = one child, 2 = two or more children, 3 = three or more children).

#### Caregiving specific factors

Those who positively endorsed caregiving status were asked “Who is the main/only person you care for?” (1 = parent, 2 = partner, 3 = child, 4 = other family member, 5 = a friend, 6 = other) and “What is the main reason the person you care for needs help?” (1 = old age or frailty, 2 = dementia, 3 = terminal illness, 4 = long term illness, 5 = learning disability or difficulty, 6 = autistic spectrum disorder, 7 = mental health problems, 8 = sight or hearing loss, 9 = physical disability, 10 = alcohol or drug dependency, 11 = other). Due to low endorsement of alcohol or drug dependency (n = 1), options 10 and 11 were collapsed into a single category. Caregiving hours were derived from the question “If so how many hours a week?” (1 = 9 h a week or less, 2 = 10 to 19 h a week, 3 = 20 to 34 h a week, 4 = 35 to 49 h a week, 5 = 50 or more hours a week).

### Data analysis

In the first stage of the analyses, descriptive statistics were produced to determine the prevalence of unpaid caring for the overall sample, and to describe the number of hours a week provided by caregivers, the relationship to the care recipient and the reason for the care recipient requiring care. Chi-square tests of independence between these variables and gender were then computed. Next, unadjusted binary logistic regression analyses were conducted to identify correlates of caregiving status. Associations are reported as odds ratios (ORs) with 95% confidence intervals.

The second stage of analysis aimed to test if there was an overall association between providing any unpaid caring and mental health and mental health help-seeking status. Two unadjusted logistic regression analyses were conducted, first with depression or anxiety and then with help-seeking as the outcome variable, and both models having caring status as the predictor variables. Associations are reported as odds ratios (ORs) with 95% confidence intervals.

In the final stage of analysis, two sets of logistic regression analyses were conducted for those who identified as unpaid carers. The first set of analyses looked at the association between demographic and household-related factors and the likelihood of meeting clinical caseness for depression or anxiety, and help-seeking. Two models were specified; the first model included all covariates separately to examine the bivariate associations between covariates and the outcomes of interest, while the second model included all covariates simultaneously to examine the association between covariates and the outcomes of interest while statistically adjusting for the potential influence of other covariates. The second set of analyses looked at the unadjusted associations between caregiving specific factors and likelihood of meeting caseness for depression or anxiety, and help-seeking. Again, two models were specified; the first model included all covariates separately to examine the bivariate associations between covariates and the outcomes of interest, while the second model included all covariates simultaneously to examine the association between covariates and the outcomes of interest while statistically adjusting for the potential influence of other covariates. Associations are reported as odds ratios (ORs) and adjusted odds ratios (AOR) with 95% confidence intervals. No variables had missing data.

## Results

### Descriptives

Of the total sample, 14.9% (n = 417) reported providing some form of unpaid care. Of those who positively endorsed caring status, 41.5% (n = 173) provided 9 h of care a week or less, 18.2% (n = 76) provided 10 to 19 h of care a week, 11.3% (n = 47) provided 20 to 34 h of care a week, 11.8% (n = 49) provided 35 to 49 h of care a week, and 17.3% (n = 72) provided 50 h or more of care a week. There were no significant gender differences in terms of hours of care provided, *χ*^*2*^ (5) = 3.08, *p* = 0.69.

The majority (42%; n = 175) of individuals who positively endorsed caring status were taking care of a parent, followed by a spouse (25.7%; n = 107) and a child (15.8%; n = 66). A small proportion of participants provided care for a different family member (1.4%; n = 40), a friend (3.4%; n = 14), or someone else (3.6%; n = 15). Although no significant gender differences were observed for relationship to care recipient, *χ*^*2*^ (5) = 6.97, *p* = 0.22, inspection of adjusted standardized residuals demonstrated that females were more likely to be a carer for a child (adjusted standardized residual= 2.6).

Regarding primary reason for requiring care, the most common reasons were old age or frailty (28.5%; n = 119), physical disability (15.1%; n = 63), long term illness (14.1%; n = 59) and dementia (9.6%; n = 40). Alcohol or drug dependency (0.2%; n = 1), sight or hearing loss (2.2%; n = 9) and learning disability or difficulty (3.4%; n = 14) were the least frequent reasons for providing care. No significant gender differences were observed for primary reason that carers were providing care for the care recipient, *χ*^*2*^ (10) = 16.58, *p* = 0.08. However, inspection of adjusted standardized residuals demonstrated how males were more likely to provide care for an individual with dementia (adjusted standardized residual = 2.9) and females for an individual with autism spectrum disorder (adjusted standardized residual = 2.2).

### Predictors of carer status and psychological wellbeing of unpaid carers

As demonstrated in Table 1, significant predictors of caring status included being aged 18 to 24 or 35 to 64 years, having three or more children in the household, having three adults in the household, and income ranging from £15,491 to £38, 740. Findings demonstrated that those who identified as carers were over five times more likely to meet clinical caseness for depression or anxiety (OR = 5.55, 95% CI 2.80, 11.01) and more than two times more likely to report receiving mental health treatment (OR = 2.04, 95% CI 1.50, 2.76).

**Table 1:** Predictors of identified as an unpaid carer in the full sample (n = 2790)

	Carer status			
	N	% carers	OR	CI
**Gender**				
Female	1401	15.4%	1.08	(0.88, 1.33)
Male	1378	14.4%	*	*
**Age category**				
18–24	257	18.7%	**1.93**	(1.28, 2.93)
25–34	406	14.0%	1.38	(0.93, 2.04)
35–64	1590	16.0%	**1.61**	(1.19, 2.18)
65 +	537	10.6%	*	*
**Relationship status**				
In a committed relationship	1764	15.5%	1.12	(0.90, 1.40)
**Number of adults in household**				
1	131	13.6%	*	*
2	1527	14.8%	1.10	(0.86,1.42)
3	317	20.2%	**1.61**	(1.14, 2.26)
4 or more	182	12.6%	0.92	(0.57, 1.49)
**Number of children in household**				
0	1433	14.1%	*	*
1	351	15.7%	1.13	(0.83, 1.55)
2	278	16.5%	1.21	(0.86, 1.70)
3 or more	95	26.3%	**2.18**	(1.36, 3.50)
**Income**				
£0–£15,490 per annum	480	14.2%	1.24	(0.87, 1.77)
£15,491–£25, 340 per annum	554	18.2%	**1.67**	(1.20, 2.33)
£25,341–£38,740 per annum	596	16.9%	**1.53**	(1.10, 2.13)
£ 38.741–£57,930 per annum	573	13.6%	1.18	(0.84, 1.67)
£57,931 or more per annum	587	11.8%	*	*
**Employment**				
Employed	1729	14.8%	*	*
Unemployed	1061	15.2%	1.03	(0.83, 1.28)

Values in bold are significant at p < .05

*This is the reference group

### Sociodemographic correlates of carer wellbeing

All subsequent analyses were restricted to those who identified as carers only to identify the carer-specific risk factors of meeting caseness for depression or anxiety (see Table 2). When the unadjusted ORs were calculated, significant predictors associated with increased risk of meeting caseness for depression or anxiety included being in the younger age categories (as compared to oldest), living in households with one or more children (compared to none), and lower income. A significant negative predictor was living in households with two or three adults (compared to lone adult households). When the adjusted ORs were calculated, younger age and living in a household with one or two children were associated with increased risk of meeting caseness for depression or anxiety. Living in a household with two or more adults was associated with lower risk of depression or anxiety.

**Table 2:** Predictors of carer depression or anxiety (n = 417)

		Depression or anxiety			
	% Carers with depression or anxiety	OR	CI	aOR	CI
**Gender**					
Female	49.1%	1.01	(0.69, 1.49)	0.97	(0.62, 1.52)
Male	48.7%				
**Age category**					
18–34	81.2%	**20.37**	(7.53, 55.12)	**14.63**	(4.71, 45.42)
25–34	78.9%	**17.64**	(6.93, 44.83)	**11.56**	(4.08, 32.75)
35–64	43.5%	**3.62**	(1.75, 4.49)	**2.72**	(1.22, 6.10)
65 +	17.5%	*	*	*	*
**Relationship status**					
In a committed relationship	46.2%	0.71	(0.47, 1.06)	1.57	(0.89, 2.76)
Not in a committed relationship	54.9%				
**Number of adults in household**					
1	39.4%	*	*		
2	28.2%	**0.60**	(0.50, 0.72)	**0.49**	(0.26, 0.91)
3	31.5%	**0.71**	(0.54, 0.94)	**0.37**	(0.18, 0.79)
4 or more	34.1%	0.80	(0.57, 1.12)	**0.41**	(0.15, 1.13)
**Number of children in household**					
0	28.2%	*	*		
1	43.0%	**1.93**	(1.53, 2.43)	**2.09**	(1.07, 4.08)
2	40.3%	**1.72**	(1.33, 2.23)	**2.54**	(1.21, 5.33)
3 or more	50.5%	**2.60**	(1.72, 3.94)	1.85	(0.68, 5.06)
**Income**					
£0–£15,490 per annum	57.4%	**2.22**	(1.12, 4.41)	1.65	(0.70, 3.87)
£15,491–£25, 340 per annum	59.4%	**2.42**	(1.29, 4.54)	2.00	(0.94, 4.24)
£25,341–£38,740 per annum	47.5%	1.50	(0.80, 2.80)	1.70	(0.83, 3.47)
£ 38.741–£57,930 per annum	41.0%	1.15	(0.69, 2.23)	1.01	(0.48, 2.14)
£57,931 or more per annum	37.7%	*	*	*	*
**Employment**					
Employed	53.1%	*	*	*	*
Unemployed	42.9%	0.66	(0.45, 9.84)	0.87	(0.52, 1.46)

Values in bold are significant at p < .05

*This is the reference group

Next, predictors of mental health help-seeking were explored (Table 3). When the unadjusted ORs were calculated, significant predictors associated with increased risk of engaging in mental health help-seeking included younger age, living in household with three or more children, and lower income, while living in a household with two or more adults was associated with reduced risk. When the adjusted ORs were calculated, a significant predictor associated with increased risk of engaging in mental health-help seeking included younger age and having two or more adults in the household was associated with decreased risk.

**Table 3:** Predictors of carer mental health treatment seeking

	% carers with mental health treatment seeking	OR	CI	aOR	CI
**Gender**					
Female	39.7%	1.17	(0.79, 1.73)	1.23	(0.80, 1.91)
Male	43.5%				
**Age category**					
18–34	52.1%	**3.68**	(1.59, 9.51)	**3.68**	(1.59, 8.51)
25–34	54.4%	**4.04**	(1.80, 9.06)	**4.04**	(1.05, 6.89)
35–64	41.6%	**2.41**	(1.24, 4.69)	2.41	(0.93, 4.18)
65 +	22.8%	*	*	*	*
**Relationship status**					
In a committed relationship	38.1%	0.71	(0.47, 1.06)	0.92	(0.54, 1.57)
Not in a committed relationship	49.3%	*	*	*	*
**Number of adults in household**					
1	60.6%	*	*	*	*
2	37.2%	**0.39**	(0.24, 0.62)	**0.40**	(0.22, 0.71)
3	35.9%	**0.37**	(0.19, 0.70)	**0.33**	(0.16, 0.68)
4 or more	21.7%	**0.18**	(0.06, 0.53)	**0.17**	(0.06, 0.52)
**Number of children in household**					
None	39.2%	*	*	*	*
One	41.8%	1.12	(0.62, 2.00)	1.18	(0.61, 2.26)
Two	50.0%	1.55	(0.83, 2.90)	1.65	(0.82, 3.31)
Three or more	60.0%	**2.33**	(1.01, 5.36)	1.74	(0.71, 4.29)
**Income**					
£0–£15,490 per annum	32.4%	**1.02**	(0.38, 2.74)	0.56	(0.24, 1.30)
£15,491–£25, 340 per annum	52.5%	**1.98**	(0.85, 4.56)	1.38	(0.68, 2.82)
£25,341–£38,740 per annum	46.5%	1.07	(0.44, 2.64)	1.49	(0.75, 2.96)
£ 38.741–£57,930 per annum	35.9%	0.87	(0.32, 2.33)	0.86	(0.42, 1.78)
£57,931 or more per annum	36.2%	*	*	*	*
**Employment**					
Employed	44.9%	*	*	*	*
Unemployed	37.3%	0.73	(0.49, 1.09)	0.95	(0.58, 1.57)

Values in bold are significant at p < .05

*This is the reference group

### Carer-specific correlates of carer wellbeing

Results from the carer-specific analyses are included in Table 4. When the unadjusted ORs were calculated, significant predictors of meeting caseness for depression or anxiety were caring for a child or other family member (as compared to parent), caring for an individual with any other condition with the exception of sight or hearing loss (as compared to old age or frailty), and providing care for 10 to 19 h a week. When the adjusted ORs were calculated, only caring for an individual with dementia, a terminal illness, long-term illness, learning disability or difficulty, mental health problems, physical disability, and other remained as significant positive predictors.

**Table 4:** Care-specific predictors of meeting clinical caseness for depression or anxiety

			Depression or anxiety			
	N	% carers with depression or anxiety	OR	CI	aOR	CI
**Relationship to care recipient**						
Parent (reference)	175	42.3%	*	*	*	*
Partner	107	43.9%	1.07	(0.66, 1.74)	0.76	(0.41. 1.39)
Child	66	68.2%	**2.93**	(1.61, 5.32)	1.94	(0.88, 4.28)
Other family member	40	62.5%	**2.28**	(1.12, 4.61)	2.15	(1.00, 4.64)
A friend	14	50.0%	1.37	(0.46, 4.06)	1.39	(0.43, 4.52)
Other	15	46.7%	1.19	(0.52, 3.44)	0.77	(0.23, 2.58)
**Reason for care recipient needing care**						
Old age or frailty (reference)	119	31.1%	*	*	*	*
Dementia	40	57.5%	**3.00**	(1.43, 6.27)	**3.19**	(1.49, 6.84)
Terminal illness	17	76.5%	**7.20**	(2.20, 23.58)	**6.53**	(1.90, 22.50)
Long term illness	59	47.5%	**2.00**	(1.05, 3.80)	**2.29**	(1.12, 4.69)
Learning disability or difficulty	14	78.6%	**8.13**	(2.14, 30.86)	**5.45**	(1.27, 23.38)
Autistic spectrum disorder	23	60.9%	**3.45**	(1.37, 8.68)	2.83	(0.89, 9.06)
Mental health problems	36	58.3%	**3.10**	(1.44, 6.69)	**3.82**	(1.60, 9.18)
Sight or hearing loss	9	55.6%	2.77	(0.70, 10.91)	3.11	(0.73, 13.25)
Physical disability	63	47.6%	**2.02**	(1.08, 3.78)	**2.29**	(0.73, 13.25)
Other	37	61.1%	**3.49**	(1.61, 7.56)	**3.35**	(1.13, 4.63)
**Number of hours providing care**						
9 h a week or less	173	45.7%	*	*	*	*
10 to 19 h a week	76	59.2%	**1.73**	(1.00, 2.98)	1.77	(0.98, 3.21)
20 to 34 h a week	47	44.7%	0.96	(0.50, 1.84)	0.99	(0.49, 2.01)
35 to 49 h a week	49	61.2%	1.88	(0.98, 3.60)	1.79	(0.88, 3.64)
50 or more hours a week	72	41.7%	0.85	(0.49, 1.48)	0.74	(0.37, 1.47)

Values in bold are significant at p < .05

*This is the reference group

Table 5 demonstrates the carer-specific risk factors associated with mental health help-seeking. When the unadjusted ORs were calculated for engaging in mental health help-seeking, caring for someone with a learning disability or difficulty was the only significant positive predictors. After adjusting for all other variables, caring for someone with a with a learning disability or difficulty was associated with increased likelihood of engaging in mental health help-seeking while caring for a friend was also associated with increased risk.

**Table 5:** Caregiving-specific predictors of mental health treatment seeking

			Mental health treatment seeking			
	N	% carers with mental health treatment seeking	OR	CI	aOR	CI
**Relationship to care recipient**						
Parent (reference)	175	12.6%	*	*	*	*
Partner	107	13.1%	1.05	(0.51, 2.15)	1.05	(0.58, 1.92)
Child	66	19.7%	1.71	(0.80, 3.62)	1.09	(0.51, 2.30)
Other family member	40	20.0%	1.74	(0.71, 4.25)	1.11	(0.52, 2.36)
A friend	14	21.4%	1.90	(0.49, 7.34)	**4.32**	(1.27, 14.73)
Other	15	26.7%	2.53	(0.74, 8.64)	3.18	(0.97, 10.43)
**Reason for care recipient needing care**						
Old age or frailty (reference)	119	11.8%	*	*		
Dementia	40	5.0%	0.40	(0.09, 1.82)	0.64	(0.29, 1.43)
Terminal illness	17	17.6%	1.61	(0.41, 6.30)	1.63	(0.56, 4.73)
Long term illness	59	13.6%	1.18	(0.46, 2.98)	0.87	(0.43, 1.79)
Learning disability or difficulty	14	42.9%	**5.63**	(1.70, 18.61)	**4.19**	(1.11, 15.74)
Autistic spectrum disorder	23	26.1%	2.65	(0.89, 7.83)	2.24	(0.73, 6.88)
Mental health problems	36	19.4%	1.81	(0.67, 4.90)	1.73	(0.75, 4.00)
Sight or hearing loss	9	22.2%	2.15	(0.40, 11.36)	2.03	(0.49, 8.42)
Physical disability	63	19.0%	1.77	(0.76, 4.09)	1.33	(0.67, 2.66)
Other	37	11.1%	0.94	(0.29, 3.05)	0.78	(0.32, 1.89)
**Number of hours providing care**						
9 h a week or less	173	38.7%	*	*	*	*
10 to 19 h a week	76	46.1%	1.35	(0.78, 2.33)	1.52	(0.85, 2.70)
20 to 34 h a week	47	46.8%	1.39	(0.73, 2.67)	1.38	(0.69, 2.78)
35 to 49 h a week	49	42.9%	1.19	(0.62, 2.26)	1.22	(0.61, 2.46)
50 or more hours a week	72	41.7%	1.13	(0.65, 1.98)	1.06	(0.54, 2.07)

Values in bold are significant at p < .05

*This is the reference group

## Discussion

This novel study describes the characteristics of unpaid carers in the UK and identifies risk factors for depression, anxiety, and mental health treatment seeking in this population. Findings from the present study can be succinctly summarised. First, approximately 15% of adults living in the UK reported providing unpaid care. Second, carers were more likely to be of a younger age, have three or more children in the home, have three adults in the household, and have a lower income. Third, risk of poor mental health was highest among carers who were younger, had several children in the household, and had a lower income. Finally, risk of depression and anxiety was highest among carers providing care for a person with long-term difficulties, while risk of mental health treatment seeking was highest among those providing care for a person with an intellectual disability.

The proportion of unpaid carers (i.e., 15%) identified in the current study is much higher (i.e., 6% higher) than what was reported in the most recent UK Census [5, 6] and the 2011 UK Census (i.e., 3.6% higher) [5]. The caring question was phrased the same in the current study as it was in Census 2021 and hence, the higher proportion of unpaid carers in the present study is likely unrelated to item phrasing. Considering that there are suggestions that the true number of unpaid carers in the UK is far greater than what was recorded in Census 2021 likely due to unpaid carers being unable to visit the homes of people they were caring for during the pandemic and some having stopped caring due to death of recipient from COVID-19 [19, 23], the higher rate in this study is not surprising. Moreover, it is widely established that many unpaid carers fail to identify as carers. According to the State of Caring Survey 2022, half of all carers took over a year to identify themselves as a carer, while more than a third of carers took 3 years [19]. Consistent with the intensity of caregiving patterns observed in Census 2021 [5], the highest proportion of unpaid carers in the current study provided less than 9 h of care per week, with over 50 h of care being most common after that. Almost half of the sample reported providing care for a parent, while females were shown to be more likely to care for a child. When it comes to providing care for a child who has a chronic illness, mothers often take a more active role than fathers do [24]. However, it was surprising that men were more likely to take care of someone with dementia. This may be attributed to the ageing population as well as the fact that women have a much greater risk of developing dementia in their lifetime than males [25].

In contrast to a recent nationwide survey which indicated that few people (i.e., 3%) under the age of 34 years identified as caregivers [19], our study found that younger age was associated with carer status. The estimates from the current study are higher than those from another recent study [26] that examined 11 waves of the nationally representative UK Household Longitudinal Study (2009–2021) and found that approximately 9% of people between the ages of 16 and 29 provided care and that this prevalence was stable between 2009 and 2021. Although the higher estimations are difficult to explain, it is likely that a greater proportion of carers fell into the younger age group in the current study because the sample was not age-representative of the UK population. Nevertheless, the high proportion of young caregivers in the current study underscores the need to raise awareness and understanding of young adult caregivers [27].

In accordance with most of the literature [13, 14, 28], findings demonstrated that compared to non-carers, unpaid carers were more likely to fulfil clinical caseness for depression or anxiety. Further underscoring the psychological burden experienced by unpaid carers [15, 29], this study showed how unpaid carers were more likely than non-caregivers to have engaged in mental health treatment seeking. Findings demonstrated how younger age was significantly associated with risk of depression or anxiety and mental health help-seeking. This is not surprising given that young adult carers are in the minority when compared to other unpaid carers and are developmentally not at a life stage to cope with the types of demands involved in such a role. Coupled with other responsibilities such as college attendance, young adult caregivers experience clinically significant levels of emotional distress [30]. Moreover, combined pressures of caring and poverty leave young people conditioned to expect less from life and tend to miss out on the opportunities and experiences that their peers have [31]. This is also consistent with the wider literature where rates of mental health problems are highest in young people [32]. Having more than one child in the household was also significantly associated with poor mental health outcomes. It is becoming increasingly common for caregivers to provide care to an older relative and their own children or grandchildren simultaneously, and these individuals are described as “sandwich generation caregivers” [33]. Research has shown how these “sandwich generation caregivers” report more emotional difficulties than non-sandwich caregivers as well as higher caregiver role overload [33]. Moreover, sandwich caring is one of the strongest predictors of poverty, with sandwich carers having a 38% poverty rate as compared to the collective carer population who have a 28% poverty rate [34]. Similarly, lower income was associated with both increased likelihood of having clinically significant depression or anxiety and mental health help-seeking. This is not surprising given that research shows unpaid carers to have a significantly higher poverty rate (28%) than non-carers (17%) [31], with the socioeconomic gradient in mental health being widely established [35].

The current study also explored caregiving-specific factors associated with risk of depression or anxiety and mental health treatment seeking. Findings suggested that the reason the care recipient needed care was significantly associated with risk of depression or anxiety over and above the relationship to the care recipient. Consistent with the extant evidence base [16, 17), caregivers providing caring for individuals with long-term difficulties (i.e., terminal illness, long-term illness, learning disability or difficulty, mental health problems, physical disability, and other) were at greatest risk of depression or anxiety. It may be that carers perceive a lack of choice in taking on the caregiving role when there are more demanding caregiving circumstances or more debilitating conditions and that this increases risk of poorer psychological wellbeing [36]. Moreover, when caring for someone with long-term difficulties, the prospect of facing their death may be a significant concern. Research has shown how carers often commence their grieving experience long before the physical death of the care recipient, a phenomenon known as anticipatory grief [37]. Research has shown how higher levels of grief and low levels of preparedness during the caring experience are associated with worse bereavement outcomes [38]. Surprisingly, there was no association between number of hours of caring and risk of depression or anxiety and mental health treatment seeking. This is difficult to explain, but it may be that more hours of providing care is not a strong predictor of maladaptive outcomes when considered within the context of other caregiving-related factors.

Only those caring for an individual with an intellectual disability were more likely to engage in mental health treatment-seeking. This is difficult to explain, however, previous research has shown how intellectual disability carers are more likely to care for multiple care recipients, to care for longer hours, be a long-term carer, provide more personal care than other carers, provide more physical care than other carers, and to experience greater financial difficulties [39]. All these factors may create a greater need to acquire mental health support. Interestingly, our findings demonstrated how caring for a partner, child, or other family member, as well as caring for someone with a long-term illness, physical disability, or other were associated with a decreased likelihood of engaging in mental health treatment seeking. Thus, this highlights the caring-specific factors which may prohibit a carer from acquiring the necessary supports.

Our study findings should be interpreted in light of several limitations. First, the data were cross-sectional, thus it is not possible to draw inferences regarding causality. Second, the current study did not assess length of caring which may have been an influential factor in determining carer status. Third, the current study did not explore whether the care recipient had any respite opportunities or whether they were in receipt of any formal supports to buffer the burden. Research has shown how carer supports reduce the likelihood of poor mental health and improve life satisfaction in carers [40]. Fourth, although the current study investigated a wide range of demographic, household and caregiving factors associated with caregiver mental health, there are likely other factors which have not been accounted for. For instance, prior research has shown how the increased risk of depression and anxiety is only present in unpaid carers experiencing conflicts with work, leisure time, and family or friends due to their unpaid caring [41]. These noted, this study’s findings have weight since they indicate that the prevalence of unpaid carers may be substantially higher than current estimates, identify the characteristics of people who are more likely to identify as unpaid carers, and evaluate the likelihood that poor mental health outcomes will arise from factors relating to both the carer and the care recipient. Fifth, the wide confidence intervals from the regression analyses indicate that the findings should be interpreted with caution. Finally, the current study was unable to explore the impact of the COVID-19 pandemic specifically on the mental health of unpaid carers. Research has shown how the COVID-19 pandemic negatively impacted carers in terms of their psychological wellbeing, difficulties in accessing social supports, concerns regarding being quarantined or care recipient being quarantined, financial worries, and treatment disruptions [42].

In conclusion, this study demonstrates how one in eight people in the UK provide unpaid care, these individuals are more likely to be younger, have multiple children in the household and have a lower income, and are more likely to experience mental health problems and be in receipt of mental health treatment. The evidence from the current study provides insight into potential public health responses to address the needs of unpaid carers including: (1) more awareness around carers’ mental health, (2) better identification of unpaid carers and their mental health needs, and (3) ensure that the mental health needs of young carers are assessed and supported. Research shows how proper respite support is associated with improved carer mental health [40], and thus this should be considered as a potential public health response to help alleviate the burden on unpaid carers. Finally, future studies should explore issues that can complicate and exacerbate poor mental health for unpaid carers, such as poverty.

## Supplementary Information

Below is the link to the electronic supplementary material.

Supplementary file1 (DOCX 16 KB)

## Data Availability

Neither the data nor the materials have been made available on a permanent third-party archive; requests for the data or materials should be sent via email to the final author (m.shevlin@ulster.ac.uk).
