# Supplementary material for: The prevalence, characteristics, and psychological wellbeing of unpaid carers in the United Kingdom
Source: Soc Psychiatry Psychiatr Epidemiol. 2024 Aug 10;60(4):869–79. doi: 10.1007/s00127-024-02745-8 (PMC12031964; doi:10.1007/s00127-024-02745-8)
Supplement: Supplementary file 1 — Supplementary file1 (DOCX 16 KB) [file 127_2024_2745_MOESM1_ESM.docx]

Supplementary Table 1: Demographic characteristics of sample

|  | N | % |
| --- | --- | --- |
| **Gender** |  |  |
| Male | 1378 | 49.4% |
| Female | 1401 | 50.4% |
|  |  |  |
| **Age** |  |  |
| 18-24 | 257 | 9.2% |
| 25-34 | 406 | 14.6% |
| 35-44 | 463 | 16.6% |
| 45-54 | 574 | 20.6% |
| 55-64 | 553 | 19.8% |
| 65+ | 537 | 19.2% |
|  |  |  |
| **Ethnicity** |  |  |
| White British/Irish | 2405 | 87.7% |
| White non-British/Irish | 101 | 3.2% |
| Indian | 63 | 2.0% |
| Pakistani | 36 | 1.3% |
| Chinese | 22 | 1.0% |
| Other ethnic group | 163 | 5.9% |
|  |  |  |
| **Highest Qualification** |  |  |
| No Qualifications | 88 | 3.2% |
| O-level / GCSE or similar | 527 | 18.9% |
| A-level or similar | 514 | 18.4% |
| Technical qualification | 351 | 12.6% |
| Undergraduate degree | 766 | 27.5% |
| Diploma | 122 | 4.4% |
| Postgraduate degree | 387 | 13.9% |
| Other qualifications | 35 | 1.3% |
|  |  |  |
| **Employment Status** |  |  |
| Employed | 1729 | 62.0% |
| Unemployed | 1061 | 38.0% |
